# Glaucoma – a neurodegenerative disease with cerebral neuroconnectivity elements

**DOI:** 10.22336/rjo.2022.43

**Published:** 2022

**Authors:** Alina Mihaela Neacșu, Dumitru Ferechide

**Affiliations:** * Ophthalmology Department, County Emergency Hospital, Brăila, Romania; ** Physiology Department, “Carol Davila” University of Medicine and Pharmacy, Bucharest, Romania

**Keywords:** neurodegenerative disease, lateral geniculate bodies, central visual pathways, neuroconnectivity

## Abstract

Glaucoma is one of important causes of irreversible vision loss and remains a global health problem.

The pathophysiology of glaucoma involves several etiopathogenic mechanisms that have generated the development of topical and surgical treatments with an effect in slowing the progression of the disease.

In the last decade, it was concluded that glaucomatous optic neuropathy is a disease with neurodegenerative elements, the destructive neuronal lesions being located not only in the structure of the retina, but also at the central visual pathways.

This review highlights the experimental and clinical data obtained so far, which underline the neurodegenerative character of the glaucomatous disease and the elements of neuroconnectivity developed during the evolution of the disease.

## Introduction

Glaucoma is an eye disease that leads to irreversible vision loss, which will affect 112 million in 2040 [**1**]. An important percentage of patients over 40 years old are affected by this pathology, and the ratio increases in people over 70 years old to 5% and in those over 80 years old to 10%. The most common form of glaucoma is the primitive open-angle glaucoma. Primitive open-angle glaucoma is characterized by increased intraocular pressure (IOP) due to impaired trabecular meshwork, that will determine the destruction of the retinal ganglion cell (RGC) axons that make up the optic nerve. After this destruction, a concentric, progressive loss of the visual field will appear. Glaucoma is an insidious disease that remains asymptomatic for many years. Visual manifestations appear when RGC axons have a permanent destructive damage, the retinal ganglion cell being unanimously recognized as the site of primary injury in glaucoma disease.

If initially it was considered that glaucoma is a multifactorial optic neuropathy, with the slowly progressive destruction of the axons of the retinal ganglion cells, resulting in the irreversible decrease of visual acuity and the visual field, starting with the year 2000, the notion of neuropathy was replaced by the neurodegenerative disease of the central visual pathways. There was a need for a new classification of glaucoma after clear evidence: despite well-controlled IOP, patients continue to lose visual acuity, suggesting that IOP-independent mechanisms are involved in progression. Current treatments are mainly aimed at lowering IOP, as the only quantifiable element easy to assess and follow, but numerous studies highlight that glaucoma progression appears in 15-25% of the patients, despite adequate IOP control [**2**,**3**], which has generated the hypothesis that there are additional mechanisms independent of IOP that are involved in glaucoma progression [**4**].

## Glaucoma – a neurodegenerative disease?

Gupta N and Yucel YH [**5**] were among the first who elaborated the hypothesis that the elevated IOP and the destruction of the RGC could trigger transsynaptic degeneration in the lateral geniculate bodies (LGB) and visual cortex. Lowering IOP is a great strategy to prevent RGC death and may decrease the risk of CNS degeneration in glaucoma. In 2009, Gupta N showed that patients with glaucoma have degenerative lesions in the LGB of the thalamus, in the magnocellular, parvocellular, and koniocellular layers. These changes were related to intraocular pressure and the severity of optic nerve injury. This hypothesis was based on in vivo MRI evidence of LGB degeneration in glaucoma, being correlated with neuropathological studies in primates and humans. Thus, the LGB atrophy could have become a relevant biomarker of visual system damage and/ or progression in some glaucoma patients. There is evidence that glaucomatous neuropathy affects the retina and the central nervous system [**5**,**6**,**7**,**8**]. Over time, increased IOP, neuronal loss reduced metabolic activity, and lesions in the expression patterns of several markers of synaptic plasticity occur in the lateral geniculate body and visual cortex of glaucoma patients and primate models [**9**]. The extension of the neurodegenerative aspect at the central visual pathways can disrupt the processing of information from the eye and allow the alteration of other neural structures of the visual process both directly and through alternative pathways that favor the processing of visual information.

Viewed as a brain disease with a neurodegenerative character, rather than a simple eye disease, the notion of glaucoma has generated numerous studies. As stated by Jeffrey L Goldberg in 2010, researches demonstrated that the complex connection between the eye and the brain is an important key to the disease [**10**].

Visual dysfunction in glaucoma results primarily from RGC death with axonal degeneration in the central nervous system [**5**,**6**,**8**,**11**]. Comparatively, in neurodegenerative disorders, the destruction of specific neuronal groups is found. While in Parkinson’s disease, the selective loss of nigrostriatal dopaminergic neurons manifests as progressive movement disorders [**12**], the loss of hippocampal and cortical neurons [**13**] translates into memory and cognitive impairments in Alzheimer’s dementia. Apoptosis, a form of programmed cell death, is associated with neuronal destruction in glaucoma and other brain diseases. Although the triggering mechanisms remain unknown, several pathological processes leading to apoptosis have been linked to mitochondrial dysfunction, oxidative stress, the release of inflammatory mediators, glutamate excitotoxicity, and abnormal protein accumulations [**14**,**15**], a form of programmed cell death. It is not known whether all these mechanisms act independently or together, but they are also mechanisms involved in Alzheimer’s dementia and Parkinson’s disease [**16**,**17**]. Amyloid β protein deposits, synuclein, and pTau have been identified in the retina of glaucoma patients. Like neurodegenerative diseases, glaucoma produces neuroinflammatory reactions, especially at the level of glial and microglial cells [**18**], the release of proinflammatory cytokines (IL1, IL6, TNFα) and chemokines (CCL2, CX3CL1) at the first central relay level (thalamus: lateral geniculate bodies and superior quadrigeminal colliculi) [**4**]. A shrinkage and loss of neurons [**19**,**20**], reduced metabolic activity [**21**,**22**], and dysfunction in the expression patterns of several markers of synaptic plasticity [**9**] in the lateral geniculate bodies and visual cortex appear in glaucoma disease and experimental primate models, after a period of increased IOP.

The most important process in glaucoma is the loss of specific neuronal populations like in other neurodegenerative diseases such as Alzheimer’s dementia (AD) [**23**]. 

Etiopathogenic mechanisms of neurodegenerative diseases are also present in glaucoma. The role of mitochondria in the process of axonal destruction is very important: through the oxidative phosphorylation pathway, mitochondria are the basic element in the synthesis of adenosine triphosphate (ATP). Organ dysfunction in oxidative phosphorylation, with electron leakage, results in a decrease in ATP production and an increase in the synthesis of reactive oxygen species: superoxides, hydrogen peroxides, and hydroxyl radicals, hyperreactive with DNA and protein structures. Free radicals in reaction with nitric oxide form peroxynitrite, determine protein nitration with modification of tyrosine residues and synthesis of nitrotyrosine [**24**]. Nitrotyrosine is an oxidation product present in many neurodegenerative diseases. Free radicals influence mitochondria [**24**], membrane potential, and Ca homeostasis, releasing pro-apoptotic factors that trigger caspase activation, a central component of apoptosis. In glaucoma disease, the generation of free radical-modified proteins can be demonstrated along the optic nerve, nitrotyrosine-modified proteins are increased in vascular walls, and levels of physiological antioxidants and superoxide scavengers are significantly reduced [**7**,**14**,**25**]. Damage to mitochondria increases the sensitivity of neurons to excitotoxicity and favors cell death resulting from excessive stimulation of neurons by excitatory amino acids such as glutamate, the major neurotransmitter in the brain [**26**]. Loss of homeostasis, like the activation of NMDA channels, increases the influx of toxic calcium ions. Excitotoxicity is an important mechanism in many brain diseases, including Parkinson’s and Alzheimer’s dementia [**26**], in which intracellular and/ or extracellular accumulation of pathologically modified proteins is present.

## Cerebral Neuroimaging in Glaucoma

The idea that glaucoma is a neurodegenerative disease that affects the central nervous pathways has opened new perspectives for this condition from a neuroimaging point of view. Studies using state-of-the-art MRI machines track the appearance and size of the lateral geniculate bodies and visual cortex in glaucoma patients. Mean diffusivity (MD) and fractional anisotropy (FA) are two parameters used in neuroimaging and offer information about brain structural integrity. MD describes the rotationally invariant magnitude of water diffusion in brain tissue. MD is a nonspecific but sensitive parameter affected by many diseases that affect barriers limiting water movement, like cell membranes. FA is a parameter that indicates the general directionality of water diffusion, which is increased in white matter tracts and lower in CSF and disorganized fibers. MD and FA have been investigated in glaucoma and showed variable correlations with RNFL thickness measured by OCT [**27**,**28**,**29**,**30**]. Advances in diffusion tensor imaging (DTI) neuroimaging allow network evaluation in vivo by integrating brain morphology with information regarding underlying tissue integrity and anatomical connectivity [**31**]. In 2009, Garaci et al. showed an affecting of these two parameters, MD and FA in glaucoma, which showed that these imaging changes could help to evaluate the severity of the glaucomatous disease [**32**]. 

MRI can show degeneration of central visual pathways after damage to RGC axons [**33**]. Degeneration of the lateral geniculate nucleus, genicular-cortical projections, and cortical areas themselves, have been explored in patients with glaucoma. Evidence of LGB atrophy has been recounted in some glaucoma patients (Gupta et al. 2009) [**34**]. Magnetization transfer imaging is a sensitive MRI technique that suggests demyelination of the genicular-calcarine zone and degeneration of the striatal zone in open-angle glaucoma, and other studies showed an increase of hyperintensities in white matter, which highlighted a cerebrovascular impairment that could have an important role in glaucoma etiopathogenic mechanisms (Kitsos et al. 2009; Zhang et al. 2016) [**35**,**36**]. In a study combining OCT and multimodal MRI, Murphy et al. found that the retinal nerve fiber layer thinning, optic nerve head changes and reduced visual cortex occurred before patients presented a visual field impairment [**37**].

The exploration of these neurodegenerative effects at the central cortical level allowed the question whether such central changes have a primary or secondary character. Early detection can be performed if central changes precede optic nerve head or retinal changes and if cerebral evaluation is more sensitive than the retinal one. Based on the data evaluating the optic nerve head and retina, in 2021, Beykin et al. [**33**] argued that the brain modifications are mainly secondary effects of anterograde and even trans-synaptic axonal degeneration. The sensitivity of different structural or functional evaluation tests of the complete visual pathway might elucidate eye-brain interactions according to disease severity, the metabolic transformations in the central visual system in glaucoma, and the utility of such measures to detect or stage the disease (Kasi et al. 2019) [**38**].

The new imaging approach in glaucoma findings highlights structural changes, but at the same time opens new perspectives in terms of identifying some clinical and behavioral effects of central origin that may appear in patients with glaucoma [**38**].

MRI is a noninvasive investigation method that favors the evaluation of several parameters at the brain level for patients with glaucoma. Neuroimaging has allowed the establishment of some evidence suggesting that glaucoma may be associated with changes in the brain [**39**,**40**]. The causes, and clinical and behavioral effects are still unknown. Glaucoma patients are at an increased risk of falling. To process the information needed for sensory integration or for balance and mobility, the brain requires attention-related information, which may be altered in glaucoma (Nuzzi et al. 2018) [**41**]. Patients with severe glaucomatous damage have difficulty maintaining balance in certain difficult postural conditions. These can be attributed to changes in brain connectivity generated by the attentional control of standing balance (Cham et al. 2018) [**42**]. This information is essential to obtain a clear idea of the central mechanisms of visual and motor deficits in glaucoma and may favor the appearance of changes related to postural difficulties.

Several diffusion tensor imaging (DTI) studies have demonstrated cortical changes consisting of degeneration of specific brain regions and white matter bundles in glaucoma patients [**32**,**43**]. It is hypothesized that the mechanism underlying brain involvement in glaucoma is supported by structural damage with a functional change [**44**]. 

Frezzotti et al. demonstrated that the interconnection of certain brain regions is associated with the severity of disease in patients with glaucoma. More structural brain abnormalities (compared to healthy controls) that could be detected in those patients [**39**,**45**], were identified. The studies are focusing on the cortical region and excluding the cerebellum demonstrated a deep and global reorganization of all brain activities [**46**]. Given the motor difficulties of glaucoma patients, the cerebellum is of particular interest in the evaluation of brain involvement in glaucoma [**47**,**48**]. There is a complex network of connectivity between different cortical areas, called the functional connectome [**44**]. Advanced neuroimaging techniques were at the base of studying structural, functional, and metabolic modifications in glaucoma patients, including gray matter atrophy damage and destruction of structural and functional connectivity [**43**,**46**] and metabolite concentration [**46**,**49**]. In this context, the involvement of some brain areas that are not directly responsible for the processing of visual information is beginning to appear in glaucoma [**39**,**43**,**45**,**50**], which issue the hypothesis of cerebral interconnectivity in glaucoma like a neurodegenerative disease. 

In a 2019 study, Minosse and Garaci evaluated brain reorganization type modifications in glaucoma. They showed that a trans-synaptic and neurodegenerative damage exists at the brain level. It has been shown that in glaucoma, functional brain damage is extended to several brain regions, especially in areas related to memory, attention, orientation, coordination, and face recognition. In these areas, brain activity was reduced/ increased. On the one hand, these changes are the consequence of an antero-retrograde trans-synaptic degeneration as a result of the pathological lesion, but on the other hand also due to the decrease in the inhibition that the visual areas normally exert on these areas of associative feedback [**44**]. The white and gray matter of several brain areas and the occipital lobe are affected with a severity directly proportional to the glaucomatous damage. These structures are involved in the integration of the visual function with higher functions, such as movement, memory, orientation, emotions and writing [**44**]. The study of patients with primary open-angle glaucoma showed that there are brain areas that activate or disappear in glaucoma patients. In this sense, the notion of cerebral neuroconnectivity appears as a need of the brain for permanent change depending on and according to the body’s needs.

These results showed a profound functional reorganization of the entire brain in glaucoma patients that was also reflected in network disruption and the appearance-disappearance of specific hubs compared to healthy controls and a different spatial distribution in the density of functional connectivity on long or short-term in glaucoma [**51**]. Two hub regions are absent in glaucoma patients: the gyrus right angular, situated in the anterolateral region of the parietal lobe, with the role in processing concepts rather than percepts in the perception-recognition-action interface [**52**] and the left lobule VIIB of the cerebellar hemisphere (with a role in fine motor coordination, in the inhibition of involuntary movement by inhibitory neurotransmitters) [**53**]. In contrast, three hubs were present only in glaucoma patients: the right inferior occipital cortex - the region is located in the occipital lobe, which contains the primary visual pathway [**41**], the right inferior temporal gyrus, located in the temporal lobe, a key area involved in the simple processing of the visual field [**54**] and the left lobule IX of the cerebellar hemisphere, an area considered essential for the visual guidance of movement [**55**].

In this context, the first cortical station of transmission and processing of the visual pathway is the primary visual cortex, from which information is transmitted to the parietal lobe and the temporal lobe. There, information is processed and feedback is provided to the primary visual cortex. Given that the hubs are not identified in glaucoma patients (i.e., in the parietal lobe and cerebellum), and that they belong to the secondary visual pathways and the hubs present only in glaucoma patients, are in the occipital lobe, one might assume that this reorganization reflects the interaction between neurodegeneration and functionality through compensatory mechanisms. Of these three hubs, two were situated in the right hemisphere, possibly leading to a lateralization hypothesis, which should however be tested statistically in a larger sample of patients [**44**].

 Furlanetto et al. demonstrated in a cross-sectional study conducted on 41 patients with glaucoma that there was a reduction in the determined height of the lateral geniculate bodies assessed by 3T MRI evaluation. But, those low values did not correlate with the severity of the glaucomatous disease assessed by functional and structural evaluations (visual field and OCT) [**56**].

In a 2020 study of 20 patients, using a 7T MRI, Kosior-Jarecka et al. demonstrated a decrease in volume of LGB in patients with glaucoma, but what is more interesting in this case is the finding of a decrease in the volume of the nucleus thalamic in the group without glaucoma and an increase in the volume in glaucoma patients group [**57**].

In 2020, Gracitelli et al. demonstrated in a cross-sectional study on 40 patients that there is a decrease in the surface of the occipital pole in both hemispheres in the glaucoma group [**58**]. 

The role of these studies showed that a decrease of volume with structural micro-alterations, which especially affects the white matter, is to show the neurodegenerative character of glaucoma with implications for future therapies [**41**,**59**], but also to emphasize the cerebral modifications of the cerebral networks in glaucoma [**43**,**44**].

## Conclusions

The eye is a very accessible organ for direct, high-resolution imaging and MRI data will continue to deepen the pathophysiological mechanisms in glaucoma with clinical and therapeutic implications. Any studies from the physiopathological sphere can help to formulate a better prognosis, to monitor the effects of the therapy and can influence the quality of life.

The new neuroimaging techniques and the use of high-precision devices (MRI 3T, 7T) offer new perspectives in identifying as many mechanisms as possible involved in the occurrence of glaucoma. The use of neuroimaging techniques in conjunction with other functional investigations of several brain areas opens a new stage in monitoring events at the level of eye-brain-behavior relationships throughout the natural history of the disease.

In perspective, identifying the elements underlying the glaucomatous changes in the central and peripheral visual pathways may favor new therapeutic approaches to prevent or even restore functional and structural loss in glaucoma.

**Conflict of interest**

The authors declare no conflict of interest.

**Acknowledgments**

None.

**Sources of Funding**

None.

**Disclosures**

None.
